# Safety Assessment of Camelid-Derived Single-Domain Antibody as Feed Additive for Juvenile Whiteleg Shrimp (*Litopenaeus vannamei*) Against White Spot Syndrome Virus

**DOI:** 10.3390/ani14202965

**Published:** 2024-10-14

**Authors:** Deni Aulia, Myung Woon Lim, In Kwon Jang, Jeong Min Seo, Hyuncheol Jeon, Haham Kim, Kyung-Min Kang, Abayomi Oladimeji Ogun, Sooa Yoon, Suhyun Lee, Junhyeok Hur, Tae-Jin Choi, Jong-Oh Kim, Seunghyung Lee

**Affiliations:** 1 Major of Aquaculture and Applied Life Sciences, Division of Fisheries Life Sciences, Pukyong National University, Busan 48513, Republic of Korea; damursalin@gmail.com (D.A.); conjp@naver.com (H.J.); haham7@naver.com (H.K.); abayomi.ogun@yahoo.com (A.O.O.); dbshelena@naver.com (S.Y.); su8842@naver.com (S.L.); junhyeok1999@naver.com (J.H.); 2 Joongkyeom Co., Ltd., Goyang-si 10260, Republic of Korea; ceo@joongkyeom.com (M.W.L.); jangik2001@gmail.com (I.K.J.); 3 Department of Microbiology, Pukyong National University, Busan 48513, Republic of Korea; sjmin2002@naver.com (J.M.S.); wkfkghs@naver.com (K.-M.K.); choitj@pknu.ac.kr (T.-J.C.)

**Keywords:** aquaculture, cold stress, immunostimulant, sdAbs, transcriptome, WSSV

## Abstract

**Simple Summary:**

Shrimp lack an adaptive immune system, which makes the oral delivery of antibodies a potential therapeutic strategy for treating diseases in these animals. Single-domain antibodies (sdAbs) derived from camelids against the white spot syndrome virus exhibit promising therapeutic potential for disease prevention and treatment, and they have emerged as effective feed additives in shrimp diets. The results of the present study demonstrated that the inclusion of sdAbs did not negatively affect juvenile whiteleg shrimp at the organism and tissue levels; however, it did impact molecular pathways associated with growth, cold stress, and antioxidant responses.

**Abstract:**

A six-week feeding trial was conducted to assess the safety of single-domain antibodies (sdAbs) derived from camelids against the white spot syndrome virus (WSSV) (WSSVvp28 was used as the antigen), focusing on the whole-organism responses and molecular-level changes in juvenile whiteleg shrimp (*Litopenaeus vannamei*). Five experimental diets with varying levels of sdAbs were formulated: CON (no sdAb supplementation); SDA_8.2_ (8.20% of sdAbs); SDA_16.4_ (16.40% of sdAbs); SDA_24.6_ (24.60% of sdAbs); and SDA_32.8_ (32.80% of sdAbs). In the CON diet, 450 mL of water per kg of diet (45%) was used to form a feed dough, while sdAbs were used to replace the water in the treatment diets. A total of 450 shrimp, with an initial body weight of 3.27 ± 0.02 g (mean ± SEM), were randomly distributed in 15 tanks (30 shrimp per tank; three tanks per treatment). Each tank was filled with 30 L of seawater (77 L capacity) in an indoor semi-recirculating system with a constant water flow rate of 1.2 L min^−1^. The photoperiod was maintained at 12 h of light and 12 h of dark. The water temperature, pH, salinity, and dissolved oxygen were 27.3 ± 0.1 °C, 7.61 ± 0.01, 34 ± 1 ppt, and 5.94 ± 0.04 mg L^−1^, respectively. During the feeding trial, the shrimp were fed the experimental diet (40% protein and 11% lipid) three times a day for six weeks. Following the feeding trial, an acute cold-water-temperature stress test was conducted by abruptly exposing the shrimp from each treatment to 15 °C for 4 h, down from 27 °C. The results showed no significant differences in the growth performance (weight gain, feed utilization efficiency, survival, etc.), plasma metabolites (aspartate aminotransferase activity, alanine aminotransferase activity, total protein, and glucose), or antioxidant enzymes (superoxide dismutase and glutathione peroxidase) among all the experimental diets (*p* > 0.05). In the acute cold-temperature stress test, there was no significant interaction between sdAb supplementation and temperature stress, nor any main effect from either factor, except for the main effect of temperature stress on the glucose levels, which was significantly higher in shrimp exposed to cold-temperature stress (*p* < 0.05). The next-generation sequencing of differentially expressed genes (DEGs) in the hepatopancreases of shrimp fed the CON, SDA_16.4_, and SDA_32.8_ diets, followed by Gene Ontology (GO) and Kyoto Encyclopedia of Genes and Genomes (KEGG) enrichment analyses, indicated that DEGs were significantly enriched in signaling pathways associated with growth, cold stress, and antioxidant systems. Overall, the results from conventional measurements suggest that the use of sdAbs against the WSSV may be safe for juvenile whiteleg shrimp. However, findings from the sophisticated analysis indicate that further research is needed to understand the molecular mechanisms underlying the observed changes, and to evaluate the long-term effects of sdAb supplementation in shrimp diets.

## 1. Introduction

Shrimp is a promising aquatic species and a vital source of animal protein to meet the growing demand for food from an expanding population. Whiteleg shrimp (*Litopenaeus vannamei*) is the most economically important shrimp species, accounting for over half of global shrimp production [1]. In 2022, the production of whiteleg shrimp reached approximately 6.8 million tons, an increase of 17.2% compared to 2020 [2]. However, challenges to the sustainability of shrimp culture include disease outbreaks due to the intensification of shrimp farms [3,4,5], the high prices of fish meal and fish oil [6,7], and the effects of climate change, such as changes in the level and quality of seawater, land use change, and severe climatic events [8,9,10,11].

Functional immunostimulants derived from plants, animals, microbes, algae, and yeast have been used as feed additives in aquafeeds to mitigate severe economic losses caused by infectious diseases [12,13,14,15,16,17,18,19]. While antibiotics are commonly used in aquaculture to prevent and treat disease outbreaks, their use has adversely affected animal, environmental, and human health. This includes promoting the proliferation of antibiotic-resistant bacteria, leading to the deterioration of water quality, and posing human health risks due to increased maximum residue limits (MRLs) and hazard risk quotients in fish treated with antibiotics [20,21,22,23,24].

Developing specific antibodies to combat viral infections is emerging as an effective strategy, not only for enhancing the immunocompetence and disease resistance of various aquatic species but also for reducing antibiotic use. This approach involves advanced techniques such as vaccination and monoclonal antibody production, which can provide targeted protection against specific pathogens [25,26,27,28]. The production of neutralizing monoclonal antibodies against white spot syndrome virus (WSSV) holds promising therapeutic potential for the prevention and treatment of the disease [29]. Monoclonal antibodies targeting the viral envelope protein (VP28) are effective at protecting *Penaeus japonicus* from WSSV [30]. Recombinant *Chlamydomonas reinhardtii* expressing VP28 has been proposed as a method to prevent WSSV infection in white shrimp through oral administration [31,32]. However, the high production costs, low stability, and large size are significant obstacles to developing antibodies for treating infectious diseases.

Single-domain antibodies (sdAbs) derived from camelids (the variable heavy domain of the heavy chain (VHH)) and sharks (immunoglobulin new antigen receptors (IgNARs)) possess several advantages, including minimal size, high stability, good solubility, strong affinity, low production costs, and low immunogenicity, making them a promising alternative to traditional antibodies [33,34,35]. SdAbs have become important tools for the diagnosis and treatment of various diseases [36,37,38]. The VHH demonstrates great adaptability to inhibit or neutralize pathogenic agents, facilitating the development of multifunctional VHH-based diagnostic and therapeutic molecules against zoonotic diseases [39]. The sdAbs developed from a white-spotted bamboo shark (*Chiloscyllium punctatum*) offer a robust tool with significant potential for diagnosing decapod iridescent virus 1 (DIV1) during shrimp cultivation [40].

In addition to the efficacy of sdAbs as a feed additive in aquafeeds, their safety must be ensured to prevent physiological anomalies in aquatic animals, such as poor ingestion rates, altered nutritional efficiency, reduced feed intake and growth (anorexia), modified kidney and liver function, depleted immune and antioxidant indicators, and decreased disease resistance [41,42,43,44]. Therefore, a thorough safety assessment should be conducted prior to their application in aquafeeds.

A safety assessment is defined as the evaluation of the likelihood of known or potential adverse health effects arising from human or animal exposure to identified hazards. Conventional methods for assessing the safety of feed additives in shrimp diets typically evaluate their effects on the organism and tissue levels, including the growth performance, mortality, feed consumption, hematology, histopathology, and disease resistance [45,46,47,48]. However, advanced methods to assess the safety of feed additives at the molecular level are still limited. Investigating transcriptome changes in active organs of the tested animals in response to a feed additive provides valuable insights into the biological mechanisms affected by the additive. Gene expression profiling using cell lines can help identify potential adverse effects on a molecular level.

Additionally, it is important to evaluate the physiological performance of shrimp in response to changing environmental conditions, such as temperature fluctuations, when they are fed diets containing newly developed additives. Thus, the current study aimed to assess sdAbs derived from camelids against WSSV as a promising feed additive for juvenile whiteleg shrimp. This assessment involved measuring the growth performance, metabolic changes, and stress responses to cold water temperatures, as well as analyzing the transcriptome profile in the hepatopancreas using next-generation sequencing techniques.

## 2. Materials and Methods

### 2.1. Preparation of Camelid Single-Domain Antibodies (sdAbs)

Camelid single-domain antibodies (sdAbs) (the technology for their development is currently under patent application) were obtained from Joongkyeom Co., Goyang-si, Gyeonggi-do, Republic of Korea. The procedures for sdAb preparation followed a previous method [35]. Briefly, a healthy adult camel was injected with an antigenic protein (WSSVvp28 obtained from the WSSV-infected shrimp was used as the antigen) mixed with a compound adjuvant once every two weeks for two months. Anticoagulated blood was collected, and B-lymphocytes were isolated from the immunized camel’s serum for total RNA extraction. Total RNA was extracted from approximately 10^7^ B-lymphocyte apheresis samples for cDNA synthesis. Oligomeric polypeptide primers were designed based on the single-chain, variable domain sequences of the immunoglobulin antibodies, and PCR amplification was performed to obtain the VHH gene fragments. The vector used to construct the sdAb library was the phage display vector (pUC199) (Invitrogen, ThermoFisher Scientific, Waltham, MA, USA). After transforming the DNA of the pUC119 vector into DH5α, it was amplified in large quantities to obtain a substantial amount of plasmid. The obtained plasmid from the vector was stored in a freezer and used as a vector for constructing the sdAb library. The acquired vector DNA was treated with the same restriction enzymes used for the insert, *Nco*I and *Not*I, and then concentrated using PCI and ethanol precipitation methods before use. The prepared insert and vector were mixed in a 3:1 ratio, and ligase (Elpis) and ligase buffer (Elpis) were added. After ligation at 16 °C for 12 h, the mixture was transformed into *E. coli* TG1 using the heat shock method to generate a small library (10^7^ to 10^8^) of high-titer, target-specific binders, referred to as the VHH library. The sdAbs in the VHH liquid were confirmed through SDS-PAGE, and the purification of the target protein as a single band was verified using a His-tag column. The VHH liquid containing crude protein that originated from *E. coli* was diluted with distilled water at a ratio of 1:16.4 to replace water in the feed manufacturing process. This dilution level served as the basis for adjusting the level of sdAb supplementation in the diet. Five graded inclusion levels of the diluted sdAbs were selected to reflect the general physiological dose–response in organisms.

### 2.2. Experimental Diet

Five experimental diets were formulated to be isonitrogenous (approximately 40%) and isolipidic (approximately 11%) based on the nutrient requirements of juvenile whiteleg shrimp [49]. To evaluate the safety of sdAbs in the whiteleg shrimp diet, a basal diet without sdAb supplementation (CON) was compared with four treatment diets that included sdAbs at levels of 8.20% (SDA_8.2_), 16.40% (SDA_16.4_), 24.60% (SDA_24.6_), and 32.80% (SDA_32.8_), all containing 10% fish meal. In the basal diet, water (450 mL per kg of diet) was used to create a feed dough. In contrast, the treatment diets used sdAbs as a substitute for water, with the volume of water adjusted according to the percentage of sdAb supplementation. The main protein sources included sardine fish meal, soybean meal, and poultry by-product meal. Fish oil and lecithin were used as lipid sources, while wheat flour and starch served as carbohydrate sources.

The procedures for diet preparation and storage were conducted following the method outlined previously [50]. Briefly, all powdered ingredients were weighed and mixed using an electronic industrial mixer (Vertical Blender 12 Inch 20QT VM-20, Hun Woo, Wuhan, China). Fish oil was slowly added to the mixer along with filtered tap water combined with sdAbs. The moistened mixture was pelleted through a 2 mm diameter die using a pelleting machine (SFD-GT, Shinsung Co., Gimpo-si, Republic of Korea). The pelletized diets were gently broken into small pellets by hand and dried in a laboratory drying machine (KE-010 Oven, Dongwon Industries, Seoul, Republic of Korea) at 45 °C for 16 h until the moisture content was less than 10%. After drying, the experimental diets were stored in plastic bags at −20 °C until further use.

The approximate composition of the test diets was determined following the standard methods [51]. Moisture content was analyzed using an atmospheric-pressure heating and drying method, where samples of a specific weight were heated in a dry oven (OF02G-4C, WiseVen^®^, Wertheim, Germany) at 135 °C for 3 h. The content of crude protein was determined by the Kjeldahl method (N × 6.25) after acid digestion using an autoanalyzer (2300, Foss Tecator AB, Höganäs, Sweden). The content of crude lipid was analyzed using the Soxhlet extraction method with the Soxtec system 1046 (Tecator AB, Höganäs) after freeze-drying the samples for 20 h. Crude ash was determined by incineration at 550 °C for 3 h in a muffle furnace (DAIHAN, WiseTherm^®^, Wonju-si, Gangwon-do, Republic of Korea). The feed formulations and approximate compositions of the five experimental diets for juvenile whiteleg shrimp are presented in Table 1.

### 2.3. Experimental Shrimp and Feeding Trial

The experimental juvenile whiteleg shrimp were donated by a local shrimp farm (Cheongsu Fisheries Co., Seosan-si, Republic of Korea) and transported to the Aquafeed Nutrition Laboratory at Pukyong National University, Busan, Republic of Korea, where this study was conducted. Prior to the feeding trial, the shrimp were acclimated to the experimental condition in an indoor semi-recirculating system with a constant water flow rate of 1.2 L min^−1^ of filtered seawater for two weeks, during which they were fed the CON diet.

At the beginning of the feeding trial, a total of 450 shrimp with an initial body weight of 3.27 ± 0.02 (mean ± SEM) were randomly distributed among 15 rectangular tanks (77 L capacity, filled with 30 L of seawater) at a stocking density of 30 shrimp per tank. Each tank was randomly assigned to one of the three replicates of five dietary treatments. Water quality was monitored twice daily, and the photoperiod was maintained at 12 h of light and 12 h of dark. The water temperature was kept at 27.3 ± 0.1 °C (mean ± SEM) using a water tank heater, the pH remained stable at 7.61 ± 0.01, the salinity was maintained at 34 ± 1 ppt, and the dissolved oxygen (DO) was maintained at 5.94 ± 0.04 using air stones in each tank connected to an air pump (LP-60A, Jeongsu Co., Busan, Republic of Korea).

During the experiment, shrimp were hand-fed their respective diets three times per day (09:30, 13:00, and 16:30 h) at a rate of 6–8% of their wet body weight per day for six weeks. The amount of diet provided was progressively adjusted based on the shrimp’s feed consumption by checking the leftover feed at the bottom of the tanks 30–45 min after feeding. Uneaten feed particles were dried, weighed, and used to correct feed intake. The total weight and number of shrimp in each experimental tank were measured every two weeks, and the feeding ratio was adjusted accordingly. Tanks were cleaned daily to remove uneaten feed and feces by siphoning after feeding, and the walls and bottoms of the tanks were scrubbed weekly. New filtered seawater was added to the system to compensate for the loss of water resulting from siphoning. Mortality was checked daily, and dead shrimp were promptly removed. The feeding ratio was recalculated after the removal of dead shrimp to maintain consistency in feeding.

### 2.4. Sample Collection and Analysis

#### 2.4.1. Growth Performance

At the beginning and end of the experiment, the total number and weight of the shrimp in each tank were counted and measured after a 24 h fasting period to calculate the initial body weight (IBW), final body weight (FBW), weight gain (WG), specific growth rate (SGR), feed efficiency (FE), feed conversion ratio (FCR), and survival rate (SR). After the final measurements, 10 shrimp from each tank of a similar size were returned to the tanks for acute cold stress testing, while the remaining shrimp were euthanized with 70% ethanol (Daejung Chemicals & Metals Co., Ltd., Gyeonggi-do, Republic of Korea) for analysis [52]. All shrimp were individually evaluated for their wet weight and total length to calculate the condition factor (CF), and hemolymph was collected from each shrimp for blood analysis. The parameters were calculated using the following equations:

Weight gain (WG, %) = (FBW − IBW)/IBW × 100

Specific growth rate (SGR, %/day) = [ln (FBW) − ln (IBW)]/day of feeding × 100

Feed efficiency (FE, %) = WG/total feed fed × 100

Feed conversion ratio (FCR) = Total feed fed/WG

Survival rate (SR, %) = [(number of initial shrimp − number of dead shrimp)]/number of initial shrimp × 100

Condition factor (CF) = (FBW/total length^3^) × 100

where the unit of weight is grams, and the unit of length is centimeters.

Hemolymph was collected from the abdominal cavity in the first abdominal segment using 1 mL syringes containing anticoagulation ethylenediaminetetraacetic acid (EDTA) (Bylabs, San Francisco, CA, USA) and transferred to 1.5 mL microfuge tubes. The plasma samples were separated by centrifugation at 13,000 rpm for 5 min using a centrifuge (CF-10, Daihan Scientific, Seoul, Republic of Korea). The separated plasma was then snap-frozen in liquid nitrogen and stored at −80 °C until analysis of the biochemical parameters and non-specific immune responses. Additionally, the hepatopancreases from the shrimp samples in the CON, SDA_16.4_, and SDA_32.8_ groups were collected for transcriptome analysis. This selective choice of the test diets (lowest, middle, and highest inclusion levels of sdAbs) was made intentionally to cover a general physiological dose–response in the transcriptomic changes in the tissue of whiteleg shrimp. The hepatopancreas was dissected from the cephalothorax, placed into a 1.5 mL microtube, instantly frozen in liquid nitrogen, and stored at −80 °C until further analysis.

#### 2.4.2. Quantification of Plasma Metabolites

The plasma samples were analyzed for metabolites, including aspartate aminotransferase activity (AST), alanine aminotransferase activity (ALT), total protein (TP), and glucose (GLU), using a chemical analyzer (Fuji DRI-CHEM NX500i, Fuji Photo Film Ltd., Tokyo, Japan).

#### 2.4.3. Quantification of Antioxidant Enzymes

The superoxide dismutase (SOD) and glutathione peroxidase (GPx) levels were determined in the plasma using enzyme-linked immunosorbent assay (ELISA) quantification kits (CUSABIO, Huston, TX, USA). Each standard reagent for SOD (cat. #: CSB-E15929Fh) and GPx (cat. #: CSB-E15930Fh), along with the plasma samples, was analyzed according to the manufacturer’s protocol. The standard reagents for the ELISA quantification kits were included in the kit provided by the company. Absorbance was measured with a microplate reader (AMR-100, Allsheng, Hangzhou, China) at an optical density of 450 nm. Sample measurements were performed in duplicate.

#### 2.4.4. Acute Cold-Temperature Exposure

The stress exposure test was conducted 24 h after the final weighing. A total of 10 healthy shrimp from each tank were randomly divided into two groups: one group was abruptly subjected to a water temperature of 15 °C for 4 h (acute cold stress treatment), while the other group was transferred to a tank maintained at ambient water temperature for the same duration (handling control). At the end of the exposure, the shrimp were euthanized with 70% ethanol for analysis. Hemolymph was obtained from the ventral sinus of the shrimp using a 1.0 mL syringe containing the EDTA (Bylabs, San Francisco) and transferred to a 1.5 mL microtube. Plasma samples were separated by centrifugation at 13,000 rpm for 5 min using a centrifuge (CF-10, Daihan Scientific, Seoul). The separated plasma was snap-frozen in liquid nitrogen and then stored at −80 °C until use for analysis of the biochemical parameters. The method of analysis was the same as that described above.

### 2.5. Transcriptome Analysis of Hepatopancreas

#### 2.5.1. RNA Extraction

Following the six-week feeding trial, total RNA was extracted from the hepatopancreases of three shrimps per treatment (only CON, SDA_16.4_, and SDA_32.8_) using the RNeasy^®^Plus Mini Kit (Qiagen, Hilden, Germany) according to the manufacturer’s instructions. The quality and concentration of the extracted RNA were assessed using a NanoDrop 2500 spectrophotometer (ThermoFisher Scientific, Waltham, MA, USA). High-quality RNA samples were then used for constructing sequencing DNA libraries. The complementary DNA (cDNA) libraries were constructed for 101 bp paired-end sequencing using a Truseq Stranded mRNA Library Kit (Illumina, San Diego, CA, USA).

#### 2.5.2. NGS

DNA libraries were sequenced using the Illumina NovaSeq 6000 sequencing platform, and the quality of the raw data was assessed using FastQC v0.11.7 (Babraham Institute, Babraham, Cambridge, UK) [53]. The Trimmomatic 0.38 program [54] was utilized to remove adapter sequences and improve the quality of paired-end sequences. The data from the RNA-seq are available on NCBI (No. PRJNA1167918). Clean reads of each treatment were then mapped to the whiteleg shrimp reference genome sequence (ASM378908v1) using the HISAT2 version 2.1.0 program [55] and were assembled using StringTie version 2.1.3b [56].

#### 2.5.3. Identification of Differentially Expressed Genes (DEGs)

The mapping results were used to analyze differentially expressed genes (DEGs) between treatment groups. FPKM values for known genes, obtained using the StringTie (2.1.3b), served as the basis of this analysis. Genes were selected based on the thresholds of |log2 (FoldChange)| ≥ 2 and an independent *t*-test raw *p*-value < 0.05. Gene Ontology enrichment analysis was conducted using the g:Profiler tool [57], and the gene set enrichment analysis was performed based on the KEGG database (http://www.genome.jp/kegg/, accessed on 10 October 2023).

### 2.6. Statistical Analysis

The results of this study are presented as the mean ± standard error of the mean (SEM). Data were evaluated for assumptions, including normality and homogeneity of variance, using the Shapiro–Wilk and Levene tests, respectively. The values from the six-week feeding trial were analyzed using one-way ANOVA to test the effects of the dietary treatments. When a significant treatment effect was observed, Tukey’s HSD test was employed to compare different treatments. Treatment effects were considered significant at a confidence level of *p* < 0.05.

The results from the acute cold-temperature exposure test were analyzed using two-way ANOVA to assess the interaction between single-domain antibody supplementation and temperature stress, as well as to evaluate the main effect of each factor. Statistical analyses were performed using the SAS program package (version 9.4, SAS Institute Inc., Cary, NC, USA).

## 3. Results

### 3.1. Growth Performance

At the end of the feeding trial, there were no significant differences in the weight gain (WG), specific growth rate (SGR), feed efficiency (FE), survival rate (SR), feed conversion ratio (FCR), and condition factor (CF) among the experimental diets (*p* > 0.05) (Table 2).

### 3.2. Plasma Metabolites

The levels of aspartate aminotransferase (AST), alanine aminotransferase (ALT), total protein (TP), and glucose (GLU) were not significantly influenced by the treatment (Table 3).

### 3.3. Antioxidant Enzymes

The results of the enzymes involved in the antioxidant systems of juvenile whiteleg shrimp fed the experimental diets indicated that the activities of glutathione peroxidase (GPx) and superoxide dismutase (SOD) were not significantly affected by the treatment (Table 4).

### 3.4. Acute Cold-Temperature Exposure

The results of the acute cold-temperature exposure on the plasma metabolites of juvenile whiteleg shrimp fed the experimental diets revealed no significant interaction between the supplementation of single-domain antibodies (sdAbs) and cold-temperature exposure regarding plasma metabolites (AST, ALT, TP, and GLU). Additionally, there was no significant main effect of sdAb supplementation on these parameters. Conversely, a significant main effect of cold-temperature stress was detected, indicating that the shrimp exposed to cold-temperature stress had higher GLU levels compared to those not exposed to stress (*p* < 0.05) (Table 5).

### 3.5. Transcriptome of Hepatopancreas

The hepatopancreas tissue from juvenile whiteleg shrimp fed the experimental diets, including CON, SDA_16.4,_ and SDA_32.8_, was subjected to transcriptome analysis. This analysis produced a total of 527,817,094 reads from the samples, yielding 520,423,740 filtered clean reads. The quality scores of ≥Q20 and ≥Q30 for each sample exceeded 98 and 95%, respectively, demonstrating the high quality of the reads. An analysis of the filtered reads revealed that 182,364,063 reads were mapped in CON, 156,541,832 reads in SDA_16.4_, and 181,517,872 reads in SDA_32.8_ (Table 6).

**Figure 1:**
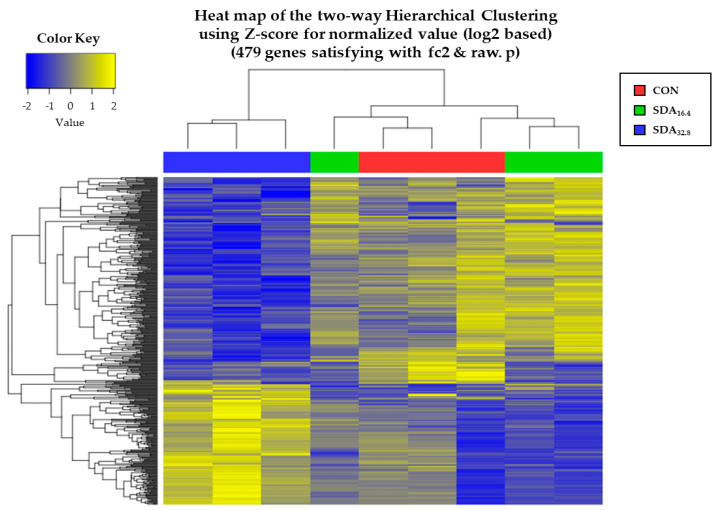
Hierarchical clustering analysis of differentially expressed genes (DEGs) between the CON, SDA_16.4_, and SDA_32.8_ groups, with yellow indicating higher expression levels and blue indicating lower expression levels.

A total of 13,235 genes were normalized using log_2_(FPKM + 1), and 479 significant genes were identified. Among these, the number of significant DEGs between CON and SDA_16.4_ was 33, comprising 11 up- and 22 down-regulated genes. In contrast, the number of DEGs between CON and SDA_32.8_ was 194, with 70 up- and 124 down-regulated genes, respectively (Figure 1).

**Figure 2:**
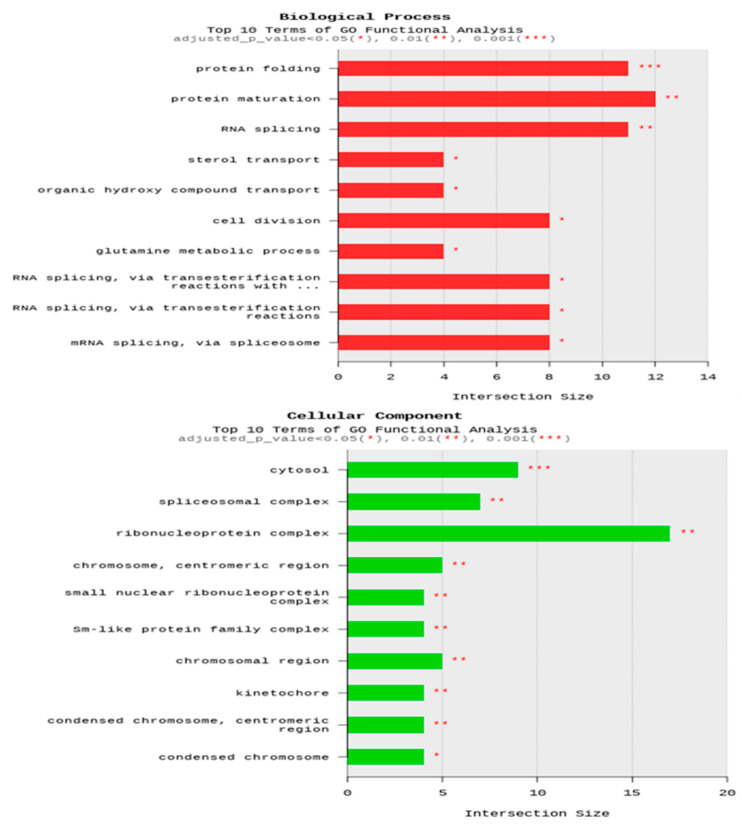

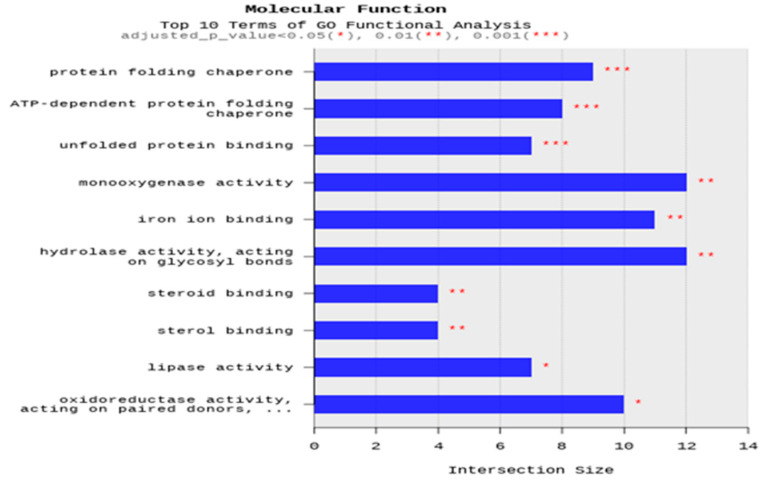
Gene Ontology (GO) enrichment analysis of differentially expressed genes (DEGs) in the hepatopancreases of juvenile whiteleg shrimp fed the experimental diets (CON, SDA_16.4_, and SDA_32.8_).

The Gene Ontology (GO) analysis categorized the differentially expressed genes (DEGs) into 514 biological processes, 119 cellular components, and 315 molecular functions, with significant enrichment observed in 60 terms (*p* < 0.05). In the biological process category, the representative terms included “protein maturation” (GO: 0051604), “amino acid metabolic process” (GO: 0006520), and “RNA splicing” (GO: 0008380). In the cellular component category, the highly represented terms were “ribonucleoprotein complex” (GO: 1990904), “cytosol” (GO: 0005829), and “spliceosomal complex” (GO: 0005681). Within molecular functions, the enriched terms included “RNA binding” (GO: 0003723), “monooxygenase activity” (GO: 0004497), and “hydrolase activity, acting on glycosyl bonds” (GO: 0016798) (Figure 2).

**Figure 3:**
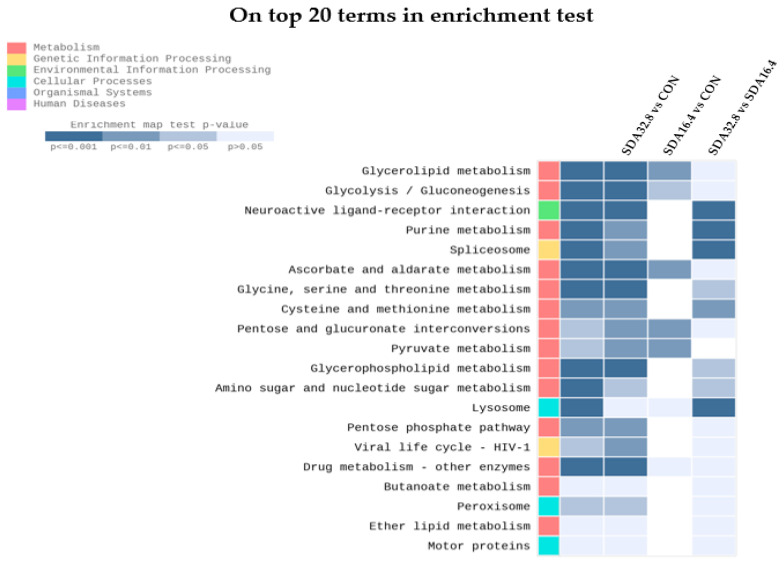
KEGG enrichment pathway of differentially expressed genes (DEGs) in the hepatopancreases of juvenile whiteleg shrimp fed the experimental diets (CON, SDA_16.4_, and SDA_32.8_).

KEGG (Kyoto Encyclopedia of Genes and Genomes) pathway analysis was used to assess the enrichment of the gene sets based on data from molecular interaction and reaction networks related to metabolism. The top 20 KEGG pathways are presented in Figure 3. The metabolism and genetic information processing pathway categories were significantly up-regulated.

## 4. Discussion

Information on the use of single-domain antibodies derived from camelids (sdAbs) as feed additives in animals, including aquatic species such as fish and shrimp, is still very limited. This study was conducted to evaluate the safety of sdAbs as a feed additive for shrimp culture. The safety assessment focused on the effect of sdAb supplementation in the shrimp diet on whole-organism responses as well as molecular-level changes. Furthermore, this study assessed the impact of the sdAb supplementation on the response to cold-temperature stress.

Growth is a crucial parameter in aquatic animal culture, signifying an increase in weight (mass) due to the accumulation of dietary nutrients in the bodies of animals. It is influenced by both the quality and quantity of the diet. Growth requires exogenous inputs from dietary sources that serve as building blocks for body components and as energy sources to maintain life processes and support the accumulation of body mass. The optimal growth of aquatic animals depends on the balance of dietary nutrients and the adequate provision of essential nutrients and energy. These nutrients, particularly the macronutrients (protein, carbohydrate, and lipid), must be balanced with dietary energy. Any imbalance among these nutrients can negatively affect growth and metabolism. Protein is the most critical nutrient and constitutes the most expensive component of fish feed, significantly supporting growth and providing energy through amino acid oxidation [58,59,60]. However, high protein levels in diets can lead to increased nitrogen release into aquatic environments, resulting in poor water quality [61]. In the present study, supplementation with sdAbs in the diet did not affect the nutritional composition, including the protein content. Furthermore, sdAb supplementation did not impact the growth performance of the juvenile whiteleg shrimp, indicating that sdAbs did not negatively affect their growth. Previous studies have shown no adverse effects of single-domain antibody administration on the body weight and survival in piglets and chickens when included in their feed [62,63].

Plasma metabolites such as aspartate aminotransferase (AST), alanine aminotransferase (ALT), total protein (TP), and glucose (GLU) are useful predictive indicators of physiological, nutritional, and immunological stress conditions in crustaceans [64]. AST and ALT are enzymes that play crucial roles in amino acid metabolism and serve as important indicators of the liver function in animals. Their secretion is positively correlated with the availability of respective substrates in the digestive system of shrimp [60]. Monitoring TP levels in plasma provides insight into an individual’s nutritional status, as changes may indicate alterations in protein metabolism associated with exogenous and/or endogenous factors. Additionally, TP examination can be useful for diagnosing the immunological status of animals [65,66]. When animals experience environmental and metabolic stress, altered GLU levels in plasma can be observed, as GLU is a key indicator of the physiological and metabolic responses to those stressors. The activation of the HPI (hypothalamic–pituitary–inter-renal) axis triggers the “fight or flight” response, leading to the release of stress hormones such as cortisol and catecholamines, which increase the glucose availability as an energy source [67,68]. In this study, the plasma levels of AST, ALT, TP, and GLU in juvenile whiteleg shrimp fed diets with sdAb supplementation did not significantly differ from the case without sdAb supplementation. These findings indicate that this supplementation did not negatively affect the physiological function or nutrient metabolism of the shrimp. Similar to our results, no changes were observed in the ALT levels in the blood of mice and rabbits treated with single-domain antibodies [68,69]. Thus, these results suggest that sdAb supplementation in shrimp diets may be used safely as a feed additive.

Whiteleg shrimp possess an innate immune system characterized by defense mechanisms that involve both cellular and humoral responses, primarily related to hemolymph, which help combat stress conditions. Superoxide dismutase (SOD) and glutathione peroxidase (GPx) are two crucial antioxidant enzymes associated with oxidative stress and immunity in crustaceans. These enzymes protect against reactive oxygen species (ROS) and free radicals, and their activities can be influenced by dietary nutrients. An increase in antioxidant defense and immune response activity indicates a disruption of the internal environment, serving as a stress indicator for animals. This suggests that greater energy is required to restore homeostasis, which can limit the growth performance by reducing the energy available for growth and metabolism [60,70]. The minimal changes in the hemolymph GPx and SOD activities in shrimp fed diets with or without sdAb supplementation indicate that the administration of sdAbs did not alter the synthesis of these enzymes or induce any oxidative stress associated with this foreign substance.

The cold-water-temperature exposure test was conducted to assess the effect of sdAb supplementation in the shrimp diet on metabolism under suboptimal conditions, and to evaluate its potential as a cold-resistant additive for protecting whiteleg shrimp from cold stress. Numerous studies have reported the use of temperature stress, including both cold and high temperatures, to evaluate the impact of feed additive supplementation in shrimp diets under suboptimal conditions. For instance, the supplementation of butyric acid (BA) in juvenile Pacific white shrimp diets effectively protects liver tissue and may regulate lipid and carbohydrate metabolism in shrimp under heat stress conditions [71]. Additionally, taurine supplementation in Pacific white shrimp diets improves lipid utilization, promotes amino acid decomposition, regulates osmotic pressure, and enhances cold resistance at low water temperatures [72]. In the present study, acute cold-temperature stress did not affect the AST, ALT, or TP levels. However, the GLU concentration was influenced by cold-temperature stress, with higher glucose levels in shrimp exposed to cold stress than those in the handling control group. Consistent with our findings, temperature did not affect the serum TP content; however, the serum GLU content of Pacific white shrimp reared at 20 °C was significantly higher than that of shrimp reared at 28 °C [72]. Similarly, the glucose concentration in the same species in the acute cold-temperature exposure group increased significantly compared to the control group, indicating the activation of the glycolysis pathway [73]. Furthermore, cold-temperature stress caused significant changes in the enzyme activity, gene expression, and metabolic product levels in Pacific white shrimp [74]. Plasma GLU is a vital energy source and can help defend against hemolymph cold stress. Increased GLU levels in hemolymph indicate stress under suboptimal conditions, such as hypothermal stress [75,76]. The rise in the GLU concentration in the juvenile whiteleg shrimp under cold stress in this study suggests a greater energy demand to adapt to low temperatures. Higher hemolymph GLU levels signify stressed physiological conditions. Importantly, the elevated GLU levels in the shrimp under acute cold stress may indicate suboptimal conditions without being influenced by sdAb supplementation in the diet. In this study, we exposed shrimp solely to cold-temperature stress; further research is needed to assess the effect of sdAbs under high-temperature conditions.

Transcriptome sequencing technology is a significant approach for quantifying the transcriptional expression in aquatic animals. In this study, Gene Ontology (GO) enrichment and KEGG pathway enrichment analyses were used to identify highly significant genes and pathways. The expression levels of genes related to growth, cold stress, and antioxidants showed significant changes at the transcript level. KEGG pathway enrichment analysis revealed that the differentially expressed genes (DEGs) were primarily enriched in metabolism pathways (glycerolipid metabolism, glycolysis/gluconeogenesis, and glycerophospholipid metabolism), genetic information processing (spliceosome), and cellular processes (lysosome). The results of the GO analysis supported these findings as well. Glycerophospholipid metabolism plays a crucial role in the recognition, signaling, and response of cells to various stimuli, combating inflammation, and synthesizing phosphatidylcholine. Furthermore, glycerolipid metabolism may promote glucose metabolism [77,78]. The spliceosome pathway is essential for regulating the response to temperature stress, including both heat and cold stress, in shrimp and fish [79,80]. Lysosomes mediate various biological processes, including plasma membrane repair, energy metabolism, maintaining metabolic homeostasis, and influencing the innate immunity of shrimp [78,81]. These results suggest that the prolonged supplementation of sdAbs beyond the duration tested in this study could influence the growth and stress response through functional changes in the metabolism and cellular processes in whiteleg shrimp. Although the inclusion of sdAbs in the diets did not demonstrate a significant effect on the growth performance, plasma metabolism, or immune response, it did impact molecular pathways associated with growth, cold stress, and the antioxidant system. Thus, we speculate that advanced analyses through transcriptome sequencing may be vital for assessing the safety of feed additives in shrimp diets.

## 5. Conclusions

In conclusion, this study demonstrated that the supplementation of single-domain antibodies (sdAbs) derived from camelids in the shrimp diet did not affect the growth performance or metabolic changes in juvenile whiteleg shrimp. However, sdAb supplementation did have effects at the molecular level related to growth, cold stress, and antioxidants. Overall, sdAbs could be considered a safe feed additive in the diet of whiteleg shrimp without negative effects at the organism and tissue levels. Further studies are needed to understand the molecular mechanisms underlying the observed changes, and to evaluate the long-term effects of single-domain antibody supplementation in shrimp diets.

## Figures and Tables

**Table 1:** Formulations and proximate compositions of the five experimental diets for juvenile whiteleg shrimp.

Ingredients	Diet				
	CON	SDA_8.2_	SDA_16.4_	SDA_24.6_	SDA_32.8_
Sardine fish meal ^1^	10.0	10.0	10.0	10.0	10.0
Soybean meal ^1^	16.7	16.7	16.7	16.7	16.7
Isolated soybean protein ^1^	7.00	7.00	7.00	7.00	7.00
Squid liver powder ^1^	5.00	5.00	5.00	5.00	5.00
Poultry by-product meal ^1^	16.0	16.0	16.0	16.0	16.0
Wheat flour ^1^	23.0	23.0	23.0	23.0	23.0
Starch ^1^	11.0	11.0	11.0	11.0	11.0
Fish oil ^1^	3.50	3.50	3.50	3.50	3.50
Lecithin ^1^	3.50	3.50	3.50	3.50	3.50
Monocalcium phosphate ^2^	0.30	0.30	0.30	0.30	0.30
Mineral Mix ^3^	2.00	2.00	2.00	2.00	2.00
Vitamin Mix ^4^	2.00	2.00	2.00	2.00	2.00
Total	100	100	100	100	100
sdAbs (dil. 16.4)	0.00	8.20	16.40	24.60	32.80
Water	45.00	36.80	28.60	20.40	12.20
Proximate composition (% of dry matter basis) of experimental diet ^5^					
Moisture	3.00 ± 0.04	3.53 ± 0.05	3.56 ± 0.05	3.84 ± 0.01	3.74 ± 0.04
Crude protein	41.3 ± 0.3	39.9 ± 0.4	41.8 ± 0.2	41.1 ± 0.3	39.9 ± 0.4
Crude lipid	12.0 ± 0.2	11.4 ± 0.0	11.9 ± 0.1	11.7 ± 0.2	11.9 ± 0.1
Crude ash	7.90 ± 0.07	7.98 ± 0.04	7.83 ± 0.02	8.07 ± 0.07	7.86 ± 0.04

^1^ The Feed Co., Goyang, Korea; ^2^ Duksan Pure Chemicals Co., Ltd., Ansan-si, Korea; ^3^ contains the following (as g/kg in premix): ferrous fumarate, 12.50; manganese sulfate, 11.25; dried ferrous sulfate, 20.0; dried cupric sulfate, 1.25; cobaltous sulfate, 0.75; zinc sulfate KVP, 13.75; calcium iodate, 0.75; magnesium sulfate, 80.20; aluminum hydroxide, 0.75; ^4^ contains the following (as mg/kg premix): A 1,000,000 IU; D 200,000 IU; E 10,000; B1 2000; B6 1500; B12 10; C 10,000; calcium pantothenic acid 5000; nicotinic acid 4500; B-biotin 10; choline chloride 30,000; inositol 5000; ^5^ values are means of duplicate samples (means ± SEMs).

**Table 2:** Growth performances of juvenile whiteleg shrimp fed different experimental diets for six weeks ^1^.

Parameters ^2^	Diets					ANOVA
	CON	SDA_8.2_	SDA_16.4_	SDA_24.6_	SDA_32.8_	
WG	98.5 ± 11.1	98.1 ± 7.5	98.7 ± 10.5	106 ± 6	97.1 ± 4.5	0.9438
SGR	1.62 ± 0.13	1.62 ± 0.09	1.63 ± 0.13	1.72 ± 0.07	1.61 ± 0.05	0.9425
FE	38.2 ± 6.8	23.6 ± 1.5	23.2 ± 10.3	31.0 ± 2.0	32.0 ± 1.3	0.3534
SR	67.8 ± 2.2	68.9 ± 4.0	64.4 ± 4.4	63.3 ± 3.3	66.7 ± 8.4	0.9228
FCR	3.25 ± 0.46	2.75 ± 0.05	2.81 ± 0.31	3.06 ± 0.22	3.06 ± 0.40	0.8036
CF	0.62 ± 0.01	0.57 ± 0.01	0.61 ± 0.01	0.59 ± 0.01	0.60 ± 0.00	0.0591

^1^ Values are means from triplicate groups of juvenile whiteleg shrimp; ^2^ WG: weight gain (%); SGR: specific growth rate (%/day); FE: feed efficiency (%); SR: survival rate (%); FCR: feed conversion ratio; CF: condition factor (%).

**Table 3:** Hemolymph biochemical parameters of juvenile whiteleg shrimp fed different experimental diets for six weeks ^1^.

Parameters ^2^	Diets					ANOVA
	CON	SDA_8.2_	SDA_16.4_	SDA_24.6_	SDA_32.8_	
AST	44.3 ± 14.7	64.3 ± 11.8	37.7 ± 6.7	105 ± 23	79.0 ± 21.5	0.0915
ALT	119 ± 32	161 ± 27	127 ± 19	167 ± 12	196 ± 22	0.2098
TP	8.07 ± 0.68	12.0 ± 1.5	9.07 ± 0.41	14.1 ± 0.9	9.83 ± 0.38	0.0650
GLU	41.0 ± 3.6	60.3 ± 5.5	51.0 ± 3.0	59.0 ± 1.0	49.3 ± 2.6	0.0658

^1^ Values are means from triplicate groups of juvenile whiteleg shrimp; ^2^ AST: aspartate aminotransferase (U/L); ALT: alanine aminotransferase (U/L); TP: total protein (g/dL); GLU: glucose (mg/dL).

**Table 4:** Antioxidant enzymes of juvenile pacific white shrimp fed different experimental diets for six weeks ^1^.

Parameters ^2^	Diets					ANOVA
	CON	SDA_8.2_	SDA_16.4_	SDA_24.6_	SDA_32.8_	
GPx	233 ± 25	231 ± 32	251 ± 46	256 ± 8	251 ± 66	0.9900
SOD	189 ± 10	303 ± 83	189 ± 14	250 ± 21	172 ± 24	0.2021

^1^ Values are means from triplicate groups of juvenile whiteleg shrimp; ^2^ GPx: glutathione peroxidase (mU/mL); SOD: superoxide dismutase (ng/mL).

**Table 5:** Effect of acute cold-temperature exposure on plasma metabolites of juvenile whiteleg shrimp fed different experimental diets for six weeks ^1^.

Treatments ^2^	Parameters ^3^			
	AST	ALT	TP	GLU
Interaction between supplementation of camelid single-domain antibodies (sdAbs) and stress exposure				
NS-CON	46.7 ± 10.1	116 ± 21	10.4 ± 0.2	55.7 ± 1.9
NS-SDA_8.2_	65.3 ± 29.8	111 ± 37	12.3 ± 2.4	53.0 ± 3.1
NS-SDA_16.4_	27.0 ± 4.0	73 ± 5	9.73 ± 0.38	47.7 ± 2.0
NS-SDA_24.6_	131 ± 57	231 ± 78	12.1 ± 2.6	53.7 ± 7.1
NS-SDA_32.8_	107 ± 68	175 ± 72	9.53 ± 0.86	49.0 ± 6.7
CTS-CON	246 ± 206	163 ± 55	9.43 ± 0.27	67.0 ± 15.6
CTS-SDA_8.2_	121 ± 76	159 ± 45	9.57 ± 0.23	74.3 ± 6.4
CTS-SDA_16.4_	135 ± 115	120 ± 78	8.37 ± 0.91	61.3 ± 3.5
CTS-SDA_24.6_	109 ± 64	148 ± 8	11.8 ± 2.6	76.0 ± 2.6
CTS-SDA_32.8_	143 ± 55	234 ± 70	11.7 ± 2.8	86.3 ± 26.8
Main effect of stress exposure				
NS	75.3 ± 19.0	141 ± 24	10.8 ± 0.7	51.8 ± 2.0 ^b^
CTS	151 ± 46	165 ± 24	10.2 ± 0.8	73.0 ± 5.9 ^a^
Main effect of dietary supplementation of sdAbs				
CON	146 ± 102	140 ± 28	9.90 ± 0.25	61.3 ± 7.5
SDA_8.2_	93.0 ± 38.5	135 ± 28	10.9 ± 1.3	63.7 ± 5.7
SDA_16.4_	81.2 ± 56.8	96 ± 37	9.05 ± 0.54	54.5 ± 3.5
SDA_24.6_	120 ± 39	190 ± 40	12.0 ± 1.7	64.8 ± 6.0
SDA_32.8_	125 ± 40	205 ± 47	10.6 ± 1.4	67.7 ± 14.9
Two-way ANOVA (*p*-value)				
Stress exposure	0.1904	0.4933	0.5616	0.0051
sdAbs	0.9488	0.2956	0.5235	0.7815
Stress exposure × sdAbs	0.7720	0.6546	0.7049	0.7656

^1^ Values are means from triplicate groups of juvenile whiteleg shrimp, where the values in each column with different superscripts are significantly different (mean ± SEM; *p* < 0.05); ^2^ NS and CTS represent the nonstress and cold-temperature stress groups, respectively; ^3^ AST: aspartate aminotransferase (U/L); ALT: alanine aminotransferase (U/L); TP: total protein (g/dL); GLU: glucose (mg/dL).

**Table 6:** Statistical summary of raw transcriptome sequencing data.

Sample ID	Total Read Bases	Total Reads	GC (%)	Q20 (%)	Q30 (%)
CON_rep1	6,091,218,898	60,309,098	46.14	98.37	95.12
CON_rep2	5,367,934,062	53,147,862	44.82	98.32	95.02
CON_rep3	5,156,550,556	51,054,956	43.63	98.42	95.26
SDA_16.4__rep1	7,211,338,390	71,399,390	46.62	98.18	94.74
SDA_16.4__rep2	5,203,115,192	51,515,992	47.67	98.27	95.0
SDA_16.4__rep3	5,347,750,424	52,948,024	46.03	98.34	95.13
SDA_32.8__rep1	5,495,075,690	54,406,690	44.45	98.17	94.76
SDA_32.8__rep2	7,640,610,408	75,649,608	43.46	98.08	94.57
SDA_32.8__rep3	5,795,932,874	57,385,474	44.94	98.41	95.27

## Data Availability

The data that support the findings of this study are available upon request from the corresponding authors. The data are not publicly available due to privacy or ethical restrictions.
